# General Medicine and Therapeutics

**Published:** 1895-05

**Authors:** 


### *SELECTIONS AND ABSTRACTS.*

### GENERAL MEDICINE AND THERAPEUTICS.

Edited by Dr. Luther B. Grandy.

### The Therapeutics of Valvular Disease.

Three points are to be taken into consideration in treating every case: (1) It should be endeavored to establish a complete compensation; (2) endeavor to cause this compensation to last as long as possible; (3) when the compensation is disturbed, it should be restored as completely as possible, and the evil effects of the disturbance speedily removed.

The author believes that a great degree of hypertrophy of the heart is not necessary for complete compensation, and that all excessive hypertrophy is a positive evil. He believes that the proper amount of hypertrophy is caused by the action of the heart in overcoming the disturbances of circulation, and that consequently gymnastics, mountain-climbing, etc., directed to the end of causing hypertrophy of the heart are preposterous. He believes the patient affected with stenosis of either orifice should be absolutely at rest in bed until the hypertrophy is attained which is necessary in each case. In the insufficiencies, which require a longer time to establish the hypertrophy of the heart, it is still more necessary to insist on such vigorous treatment during the whole time except under certain circumstances. To maintain the hypertrophy thus obtained the patient should at first be out of bed for a short time; the time is gradually lengthened, and then light passive gymnastics may be practised, and finally more severe work may be done. Each patient is a rule unto himself as to the degree of exercise to be taken, but every patient should be warned that it will be fatal for him to make attempts at hurried running, gymnastics, or mountain-climbing, and that he should forever forego such exercise.

The best rule to give to the patient is to insist that any exercise which causes palpitation of the heart or dyspnœa should not be indulged in, and that the border-line of safety is reached when the heart is caused to beat one hundred in the minute. If this rule is not followed the compensation will not be maintained for a great while. The diet is also an important element in maintaining the compensation. A mixed diet is the best in which the proteids are in excess, milk in excess with but little fat and carbohydrates. The patient may drink as much milk and mineral water as he desires. Alcoholic drinks should be taken but sparingly; coffee and tea may be taken well diluted. When there is a disturbance of the compensation, rest in bed is necessary, and by this alone the compensation may sometimes be reëstablished. If rest does not do all that is necessary, then drugs must be used; of these, digitalis is the chief. The action of digitalis is very transitory and will last but three or four weeks, and one cannot be certain of a good digitalis action unless at least four weeks have passed since its last use. He never orders digitalis unless the patient will remain in bed, or at least remain quiet until its full action is established. A dose of the infusion of thirty to sixty grains, or of the powder of twelve to twenty grains, should be used in the course of three to five days and will thus cause the full digitalis action. It is necessary that after this dose the drug should be entirely discontinued and not used in small doses. In twenty years 124 cases were thus treated, 61 had complete restoration of the compensation, 40 were improved for a short time, and 23 were unaffected. Certain cases will not respond to this treatment by digitalis; in these tincture of strophanthus may be used continuously in five to ten-drop doses three times in the day. The best diuretic is calomel in doses of three to five grains two or three times daily. Caffein may be used in the form of salicylate or benzoate of caffein and soda. Some cases respond well to hydragogue methods. Warm baths may be used, but care must be taken that the temperature of the patient is not raised above 102° for fear of cardiac paralysis. When there is much effusion into the cavities, paracentesis may be practised. In patients with very irregular heart action cold applications to the cardiac region may be tried for a short time. In other cases with heart failure, camphor and alcohol may be used. In very severe attacks of lack of compensation, bloodletting may be practised with good results. Severe pulmonary œdema or signs of œdema of the brain are indications for bloodletting, also cases which fail to respond to rest, digitalis, and the other means suggested—cases which will sometimes respond to digitalis after bloodletting that would not before.—*Univ. Med. Mag.*

---

### THE ANTITOXIN TREATMENT IN NEW YORK.

The meeting of the New York Academy of Medicine for April 3d was arranged to be a symposium on the antitoxin-treatment of diphtheria, and a number of those who had had special opportunities to study this subject practically were invited to participate in the proceedings. Dr. Herman M. Biggs opened the discussion by a historic and statistical report. The cases were divided into two classes, viz.: (1) those treated in the Willard Parker Hospital, and (2) those treated in the tenement-houses by the physicians attached to the Board of Health. In the latter class were 255 cases, with 40 deaths, or a mortality of 15.69 per cent. Fifteen of these deaths occurred within twelve hours after the injection of the antitoxin. If these were excluded, the mortality would be 10.4 per cent. Out of 197 cases in which the antitoxin treatment was begun before the fifth day of the disease, the mortality was only 7.79 per cent., and all the evidence seems to point to the fact that but little benefit can be expected from the treatment unless it is begun at an earlier stage than this. The series of hospital cases did not make such a good showing, probably owing to the fact that this list comprised many severe cases, and they did not come under observation until later on in the disease. Out of the 129 cases in this class, there were 31 deaths, or a little more than 24 per cent. Comparing these figures with those obtained from the cases treated in this hospital in the same season of 1894, we find that at that time 146 cases of diphtheria were treated without the antitoxin, with a mortality of 32 per cent. Dr. Biggs said that in his experience the effect of the antitoxin on the membrane had always been quite marked, even when there had been but little improvement in the general condition. In a little more than 6 per cent. of the hospital cases eruptions, for the most part resembling urticaria, were observed, and albuminuria was observed in about the same proportion of cases. In most instances the serum employed was that manufactured under the auspices of the Board of Health. One case had a very troublesome urticaria, followed by swelling and pain in the larger joints and by loss of power in many of the muscles. In concluding his remarks the speaker said that it should not be forgotten that diphtheria-antitoxin is a specific only against diphtheria-toxemia; it does not restore renal cells or heart-fibers already degenerated, or relieve laryngeal stenosis. The earlier the antitoxin is administered, the more striking its effect. The mortality among cases throughout the city that were not treated with antitoxin was between 25 and 35 per cent. at the same time that the cases treated with the antitoxin yielded a mortality rate of only 8 to 10 per cent. This in itself was a sufficient comment on the value of the new treatment.

Dr. C. H. Peck then described the success that had attended the use of the antitoxin in controlling a very extensive and stubborn epidemic of diphtheria in the New York Infant Asylum. The temporary immunity conferred in this way proved far more effectual than all the previous attempts that had been made to stay the disease by isolation.

Dr. George P. Biggs, after making eighteen autopsies on patients who had died after the antitoxin treatment, said that he had come to the conclusion that the antitoxin had a distinct influence in loosening the diphtheric membrane, and that it also tended to check decidedly parenchymatous degenerations of the internal organs.

After the reading of papers the general discussion of the subject was opened by Dr. Joseph E. Winters. He denounced the new treatment most vigorously, and stated that careful observations in the Willard Parker Hospital during the past three months had not only forced him to take this stand, but had taught him to consider the statistics presented in the paper by Dr. Biggs as wholly unreliable and misleading. To support this strong assertion he cited a number of cases appearing in the statistics as having been successfully treated with the antitoxin, and narrated how he had been unable to find any *clinical* evidence of diphtheria in some, and only the mildest manifestations of the disease in others, although doubtless all of them had given the bacteriologic diagnosis of diphtheria. Passing over some of the minor points made in his sweeping criticism and denunciation of the work done in this hospital by the advocates of the antitoxin treatment, attention should be called to the fact that Dr. Winters claims that the temperature-charts and many of the symptoms presented by not a few of the cases treated by the new method were strongly indicative of the existence of septicemia, and that the treatment resulted in establishing a condition of profound anemia. In his opinion the element of individual susceptibility would explain the disastrous results observed, including the fatal case very recently reported in Brooklyn. In conclusion, he said: "I oppose the antitoxin treatment because in the 150 cases treated in the Willard Parker Hospital there is not the slightest evidence that a single symptom has been relieved, and because it is dangerous in its immediate effects on the kidneys and nerve centers, and in its remote effects on the blood."

Dr. George L. Peabody detailed his experience, showing that a bacteriologic diagnosis *alone* was very often far from satisfactory, and that something else besides the Loeffler bacillus was necessary for the development of the disease. His own experience with the antitoxin treatment had been quite encouraging, and he thought that the bad results that had been observed by Dr. Winters might possibly be explained on the ground that they were cases of mixed infection, over which, it is well known, the diphtheria-antitoxin was powerless.

Dr. L. Emmet Holt expressed his confidence in the antitoxin treatment, and expressed very similar views regarding the shortcomings of the bacteriologic test. A complete diagnosis of diphtheria should be founded on both clinical and bacteriological grounds.

Dr. W. L. Somerset, of the Willard Parker Hospital, showed from the records of that hospital that there had been a distinct lowering of the mortality rate by the new treatment.

Dr. Herman M. Biggs, in closing the discussion, said that Dr. Winters had shown himself in many ways to be biased in his views on this subject, and perhaps in none more than in his allegation that there had been but little severe diphtheria in the city. It was not claimed that diphtheria-antitoxin would neutralize sepsis or anything but diphtheria-toxemia. So far as he knew, no case had been included in his statistics in which there had not been a clinical as well as a bacteriological diagnosis of diphtheria made before beginning the treatment. The fact that there had been a sudden and marked lowering of the mortality in diphtheria all over the world since the adoption of the antitoxin treatment was a sufficient refutation of the charge that it was wholly valueless.—*Medical News.*

---

### MORPHINOMANIA.

Let me warn, with all the weight I can command, every doctor who may be dallying with this drug or who may think its self-taking called for—and this warning holds with special force if the subdermic method be practiced—let me warn him that he is inviting disaster by jeopardizing interests vital to his well-being; and let me urge him to pause, and to ponder well, whether, despite this warning, he dare take such risk. Let him not be blinded by any underestimate of the poppy's power to ensnare. Let him not be deluded by an over-confidence in his own strength to resist; for along this line history has repeated itself with sorrowful frequency, and—as my experience will well attest—on these two treacherous rocks hundreds of promising lives have gone awreck. . . .

I have no wish to pose as an alarmist, but I will say that many a doctor who gives himself a daily hypodermatic dose of morphine for a fortnight will come perilously close to the danger line—beyond which, bondage begins. . . .

Regarding the over-use of morphine, never was there so little excuse for it as now, for never were the means at command to ease pain and bring sleep equal to those of to-day. Modern medicine is richly equipped in this regard, and if these resources be fully availed of it will go far to decrease this ill. . . .

Teachers in medical schools should realize that they have opportunity to wield great influence for good, and by word and deed they should improve it. To do so would strike right at the root of this evil, for I truly think the junior members of the profession are the greatest sinners in this regard; and if, by timely counsel from their preceptors and college instructors, the thousands who year after year begin a medical career can be brought to believe the danger incident to an incautious or needless giving of morphine, and then shape their practice in keeping with that belief, the good work will be largely done. . . .

More and more the oldest medical men, impelled by larger wisdom, or an experience often unhappy, are quitting the syringe; more and more rarely are they using morphine.—J. B. MATTISON, M.D., in "*Morphinism in Medical Men.*"

---

### TRIONAL IN THE INSOMNIA OF CHILDREN.

In a paper on this subject Claus concludes (*Therapeutic Gazette*):

1. Trional, in the dose of one-third to twenty-two grains, according to the age of the child, is a brilliant hypnotic. On the following morning neither headache nor heaviness of the head was noticed. Physiological sleep was favored. Patients do not become accustomed to it. Sleep occurred in ten or fifteen minutes after its administration.

2. Trional has no very pronounced effect upon insomnia the result of pain.

3. Trional leaves the intellectual, respiratory, and circulatory functions untouched, and it has a favorable effect upon digestion.

4. In toxic insomnia, particularly that caused by alcohol, chloral seems to be more active.

---

### ANTIPYRIN AS A HEMOSTATIC.

Dr. Roswell Park, in the *Medical News*, recommends a 5 per cent. solution of antipyrin in sterilized water as an efficient hemostatic. He uses it as a spray in the nasal cavity, for epistasis, upon the peritoneum, upon the surface of the brain, or wherever else there is oozing. Of course it has not power to contract vessels of any size that spurt, but will almost instantly check oozing. He has found it also to be unirritating.—*Ex.*

